# How Much Sugar is in My Drink? The Power of Visual Cues

**DOI:** 10.3390/nu12020394

**Published:** 2020-02-02

**Authors:** Bethany D. Merillat, Claudia González-Vallejo

**Affiliations:** Ohio University, Athens, OH 45701, USA; gonzalez@ohio.edu

**Keywords:** nutrition facts panel, food label, consumer behavior, food decision making, food packaging, food choice, nutrition and health claims, food label, sugar, nutrition

## Abstract

Despite widespread attempts to educate consumers about the dangers of sugar, as well as the advent of nutritional labeling, individuals still struggle to make educated decisions about the foods they eat, and/or to use the Nutrition Facts Panel. This study examined the effect of visual aids on judgments of sugar quantity in popular drinks, and choices. 261 volunteers at four different locations evaluated 11 common beverages. Key measures were estimates of sugar in the drinks, nutrition knowledge, and desire to consume them. In the experimental condition, participants viewed beverages along with test tubes filled with the total amount of sugar in each drink; the control condition had no sugar display. Both groups were encouraged to examine the Nutrition Facts Panel when making their evaluations. Correlational analyses revealed that consumers exposed to the visual aid overestimated sugar content and the length of time needed to exercise to burn off the calories; they also had lower intentions to consume any of the beverages. Individuals asserting to use the Nutrition Facts Panel (NFP) in general were also less likely to admit using it in this particular study (*r* = −2, *p* = 0.001). This study suggests that a simple visual aid intervention affected judgments and choices towards curtailing sugar intake. This has implications for labeling format implementation.

## 1. Introduction

“…sugar is cheap, sugar tastes good and sugar sells, so companies have little incentive to change [1] (p.29).” 

A U.S. citizen consumes an average of 216 L of soda per year, of which 58% contains sugar [1]. The obesity problem in the U.S. is well documented, with obesity rates as high as 25% in 41 states, and above 20% in every state [2]. Importantly, child obesity has increased in the 1999–2016 period as a recent report shows [3]. Researchers have attributed the obesity problem, in part, to consumption of nutrient poor processed foods, containing high amounts of sugar, sodium, and saturated fats [4,5]. 

Added sugars alone are linked to a wide range of non-communicable diseases, including tooth decay, gout, heart disease, diabetes, obesity, and metabolic syndrome which is characterized by higher blood pressure, blood sugar, and triglycerides, and lower “good” cholesterol [6]. The health care costs associated with metabolic syndrome is estimated to be $150 billion annually [1]. Furthermore, the United Nations World Health Organization places non-communicable diseases as the leading cause of deaths globally, responsible for about 68% of deaths worldwide in 2012 [7]. Given the relationship between added sugar consumption and metabolic syndrome, researchers have gone as far as to call sugar a toxin, and have proposed stricter regulations for added sugars similar to those controlling alcohol [1]. 

While the American Heart Association recommends that the population limit added sugar to six teaspoons a day for women and nine for men [6,7], the average sugar consumption in the form of fructose is at an all-time high with current estimates at around 57.7g/day (or approximately 14.42 teaspoons-4g per teaspoon) accounting for 10.2% of total caloric intake. A study also placed sugar-sweetened beverages as the most important contributor of fructose intake (30.1%) [8]. 

### 1.1. Strategies to Promote Healthier Eating

Increasing the availability of healthy foods, and/or restricting specific types of foods, such as soft drinks in school settings, have proven effective methods to curtail poor nutritional consumption by both the Food and Agricultural Organization of the United Nations (FAO) [9,10] and the Centers for Disease control [11] (see also [12,13,14]. Curbing choices via pricing has also proven effective [15] (see the Chilean experience in Jacobs [16]). The FAO further notes that education-only interventions appeared less successful than those including environmental changes [10]. This poses a challenge for promoting better food choices outside school settings because price adjusting and/or limiting the supply of certain foods is more difficult to implement [16].

In a positive light, a number of prominent health campaigns are targeting the consumption of sugary drinks in many states (e.g., California’s Kick the Can campaign; the Kansas’ Just Add Water! public health intervention). However, a report from the Food and Agriculture Organization of the United Nations [10] concludes that public awareness campaigns, which take many different forms all over the world, have received mixed support regarding their effectiveness.

Another strategy to encourage healthy eating is by means of nutrition labelling. To address the issue of unhealthy eating, the US Nutritional Labeling and Education Act of 1990 [17] mandated the use of a standardized nutrition label (the Nutrition Facts Panel, NFP). The aim of the law was to provide consumers with nutritional information that was accurate and easy to read and encourage healthier food choices [18]. Studies by the US Agriculture Department found that the percentage of adults who reported using the NFP ‘always or most of the time’ went from 34% in 2007–08 to 42% in 2009–10 [19] and 77% in 2014 [20]. 

However, the assumption that the NFP indeed helps consumers to judge the nutritional quality of the foods and to make better decisions is debatable. In the 2014 survey, half of those who reported rarely or never using the NFP said they did not feel they needed to use the label [20], and several studies indicate that there has been no aggregate improvement of American nutrient consumption since the implementation of the NFP [21,22]. People may think that they do not need to use the label, but their health may be suffering because with the myriad of products in the market today, understanding of, and the ability to use the NFP can make a significant difference in one’s ability to judge the healthfulness of food and drink options.

Current studies find positive and significant correlation between judgments of nutrition quality of foods based on the NFP, and a nutrition quality expert standard, but the levels of agreement are low [23,24]. More broadly, these studies and others investigating a host of other ecological factors, including those related to dietary choices, necessitate viewing the role of the NFP through the context of a multi-factored public health issue.

The FAO’s 2013 report reveals a greater understanding of nutritional information form label usage, but not necessarily improvements in consumption [10]. Additionally, nutrient lists, which is the format used by the U.S. Nutrition Facts Panel (NFP), are often found to be confusing, and may disproportionally affect individuals having lower knowledge about nutrition and health. Why? While a number of factors certainly contribute to the problem, research has found that both those of lower socio-economic status and individuals with lower knowledge concerning nutrition and health are less likely to use such labels [25]. While more generally, research shows that the process by which food marketing affects food decisions is not well understood [26], and although a number of suggestions on how to improve consumer choice have been proposed, few are supported with empirical research [27]. Further investigation of packaged label use is still needed to determine whether they have a positive effect on nutritional understanding and decision making [28].

However, there are a number of other interventions which have both real-world applicability and have been proven to improve consumer choices. For example, research by Donnelly et al. found that evocative, graphic warning labels, as compared to text warning labels (calorie labels and no labels) significantly reduced the share of sugary drinks purchased in a cafeteria [29]. These graphic labels also served to heighten negative affect (toward unhealthy options) while promoting deeper thought concerning the health consequences of consuming sugary options [29]. We point this out to highlight that for an intervention to have success in changing consumer behavior, it must be both effective in research studies, and have the capability of being implemented and accepted in the broader consumer market (including both consumers and retailers).

While these studies have certainly played an important role in our understanding of the power of visual aids, it is important to consider that they may have limited real-world applicability because of the difficulty of adopting them in the market, and making them visible/available to consumers on a wide-scale.

### 1.2. The Present Study

The central and negative role of sugar in human health has been identified in numerous sources (e.g., Williams and Nestle [30]) and in conjunction with the current debates on designing successful interventions via NFP changes [31], a direct examination of judgments of nutrients from label information is in demand. In particular, the current study contrasts perceptions of sugar content in beverages when consumers use the current NFP vs. using the NFP with the addition of a simple visual aid.

A study by Viskaal-van Dongen, de Graaf, Siebelink, and Kok [32] exemplified the importance of visualizing nutrition content in order to properly judge it. In that study, participants consumed either a meal with visibly fatty food, (e.g., bread with butter on top), or invisible fat (e.g., bread baked with extra oil). Unbeknownst to the participants, both meals contained the exact same amount of energy, fat, carbohydrates and proteins, but participants ate 9% more calories when the fat was hidden than when it was visible. Hence, judging the hidden nutritional make up of food is not simple and can lead to overconsumption. More generally, psychological research has shown that judgments are fallible in many domains [33,34].

Following the work of Viskaal-van et al. [32] we coin the term the hidden sugar hypothesis to propose that individuals are not able to make accurate judgments of sugar content in beverages because the solid sugar, like many other nutrients, is invisible or abstract, even when numerical information is available via the NFP, without any visible cues. We hypothesize that participants underestimate the amount of sugar and the number of calories in a drink when the sugar is hidden. In contrast, we expect different and more accurate perceptions when the amount of sugar is explicitly present. Better perceptions of amount would also lead to better judgments of other related variables, such as the amount of time needed to walk to burn off the calories in the drink. We also assumed that variability in judgments would relate to consumption intentions.

One aspect pertaining to the effectiveness of interventions on food consumption is nutrition knowledge. Studies have shown that greater nutrition knowledge is associated with increased intake of fruits and vegetables and greater adherence to recommendations on fat intake [35]. These researchers developed the Nutrition Knowledge Questionnaire (NKQ) and found that a lack of nutritional knowledge impacted the relationship between diet and disease (e.g., between high fat and salt intake and cardiovascular disease) [35]. Similarly, individuals high in motivation and obesity knowledge, termed the ‘nutrition elite’ were found to have appropriate evaluations of nutrient claims that impacted consumption intentions [36].

We thus measured participants on several individual-level measures including the participant’s health, nutrition knowledge, education, and other demographic information that could impact their judgments and choices. We predicted that higher scores on the NKQ, indicating greater nutrition knowledge, would be associated with more accurate ratings of the healthfulness of beverages, and more accurate estimates of the amount of sugar contained in the drinks and walking estimates. Additionally, we predicted that for individuals with diabetes, accuracy of evaluations would be greatest irrespective of the display manipulation. Following past research as reviewed above, we also expected relations of self-report of NFP usage with variables such as nutrition knowledge, education, and income.

## 2. Materials and Methods 

### 2.1. Participants

Participants (*n* = 261) were volunteers who came to shop at four different locales in Ohio. They were predominantly female (54.8%), and not currently dieting (75.1%), with 43.7% reporting that they were employed, and 6.9% were on disability. While the sample was largely Caucasian (87.70%), it also included African Americans (3.80%), Hispanics (1.10%), and Asians (2.70%). The mean age was 45.80 (SD = 17.02), and the average BMI for this group was 28.41 (SD = 6.89), which is considered overweight (BMIs 25–29.9) by the U.S. Department of Agriculture and U.S. Department of Health and Human Services Report (2010). Seventy percent of the sample reported an annual income of less than $49,000 per year. With relation to health, 37.5 % reported having health issues or other dietary restrictions that influenced their food choices. 52% reported eating out at least once a week or more, and over half the sample (58.5%) said they used the NFP 70% of the time.

Of the participants, 146 completed the survey in the sugar/tube and NPF condition (referred to as the Sugar group in what follows); these participants saw test tubes filled with the exact amount of sugar in each drink attached to each of the beverages, with the NFP visible as well. 115 participants completed the survey in the NFP alone condition (referred to as the No-Sugar group or control group). Participants in the No-Sugar condition only saw the beverage—no test tubes were attached but the NFP was visible. There were no significant differences between the two conditions for gender, dieting, employment, race, age, BMI, income, health issues, eating out or use of the NFP.

### 2.2. Procedure

For the study, the researchers set up a small folding station at five different locations in Ohio: Lottridge Ridge Food Pantry, Save-A-Lot, the Athens Farmers Market, and the Solon Community Center (Table 1). Participants were randomly assigned to the control (No-Sugar) and experimental (Sugar) groups. The stand was set on different days with randomized days to conditions so as to obtain approximately equal number of participants from each location in each experimental condition. 

**Figure 1:**
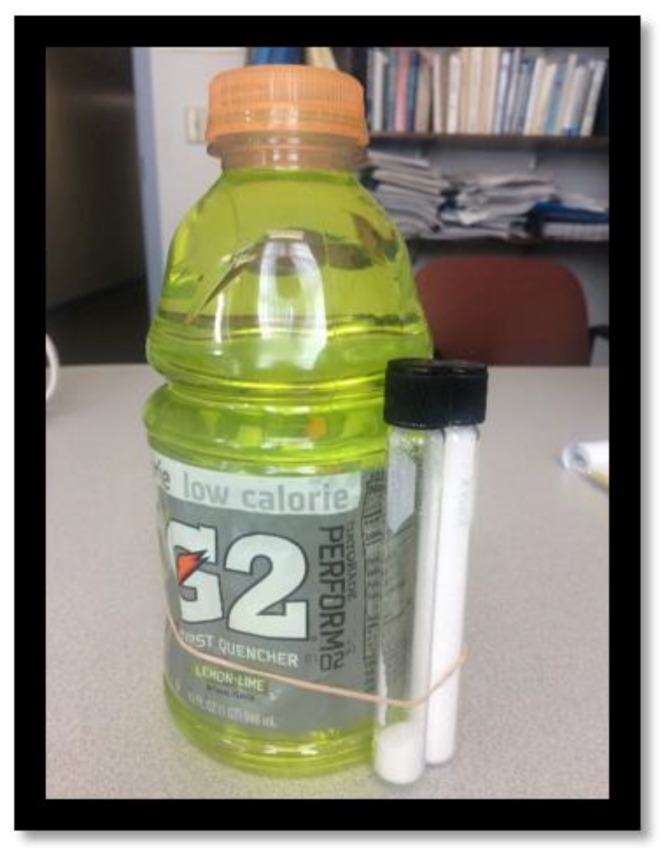
A drink with its sugar content displayed.

On the table, there were 11 popular beverages (see Table 2). In the No-Sugar condition, the beverages were presented alone; in the experimental Sugar condition, sugar bottles (test tubes) filled with the exact grams of sugar contained in the entire beverage were attached to the drink with rubber bands. Figure 1 shows one such display.

Participants were solicited for the study as they walked by the booth. They were asked if they would be willing to complete a short survey in exchange for the chance to win a $50 Visa gift card. If they agreed, they were read the informed consent statement and signed that they understood and were willing to participate. Participants were given a survey packet and writing utensil and completed the survey as they stood by the table which contained unopened bottles of all 11 drinks. They were told that they should answer the questions to the best of their knowledge, and were encouraged to use the NFP and all other information about the drinks to help answer the questions. 

Participants, after answering a series of questions for each drink, completed a demographic questionnaire, a nutrition quiz, and follow-up questions concerning their affective state and experience participating in the study. All measures are found in Supplementary Materials Figures S1–S4. After completing the survey, they were thanked for their time and debriefed. 

### 2.3. Measures

Expert Nutrition Quality Scores: NuVal^®^ is a Nutrition Scoring System developed by medical and nutritional experts which summarizes the overall nutrition of a food on a scale from 1 to 100 (with higher scores indicating more nutritious food) [37]. It utilizes an Overall Nutritional Quality Index (ONQI) algorithm to convert the complex nutritional information from the Nutrition Facts Panel into a single score. For this study, NuVal scores were used as the “gold standard” with which to determine how accurately participants could judge the healthfulness of a food. NuVal ratings for the beverages used in this study can be found in Table 2.

Beverage Questions: For each of the 11 beverages in the study, participants were asked to, “Please answer the following questions based on what you observed today at the nutrition and sugar display.” On each page of the packet, a picture of the beverage from the display was shown, along with the drink’s name, and the participant was asked to answer seven questions concerning the drink: “If you consume (drink name) how many times a week do you drink it (put 0 if you never consume it or don’t like it)?”; “What proportion of this beverage is sugar (e.g., if a drink contains ½ a cup of sugar, and ½ cup of milk, the beverage would be ½ or 50% sugar)?”; “How HEALTHY is this beverage?” (on a scale from 0 to 100, with 100 being the healthiest); “How well does the beverage meet nutritional requirements/how NUTRITIOUS is the drink?” (on a scale from 0 to 100, with 100 “meets them extremely well”); “How many teaspoons of sugar are in this drink?”; “How many minutes of brisk walking (3.5 mph) would it take to burn off the calories from consuming this drink (assume you are drinking the ENTIRE bottle, which may contain more than one serving size)?”, and “How confident are you in your answer?” (for the walking estimate). They completed all of these questions for each of the 11 drinks found in Table 2.

Demographic Questions: Participants answered a comprehensive set of questions concerning their height, weight, age, gender highest level of education and employment. Information was also gathered concerning their eating habits, medical history, and use of packaging/NFP when making purchases. Participants also rated the importance of the various nutrients in the NFP in general, and in relation to their use in this study. Finally, they were asked qualitative questions concerning their participation in the study, and factors they believed would influence their choice of healthy vs. unhealthy foods, as well as their knowledge of health guidelines.

Nutrition Knowledge Questionnaire (NKQ): The NKQ [38] was designed to provide a comprehensive measure of nutritional knowledge in adult populations. The scale consists of items concerning dietary advice, dieting and disease in five main areas: understanding of health terminology (e.g., fiber and cholesterol); awareness of dietary recommendations; knowledge of food sources related to the recommendations (e.g., which foods contain which nutrients); using dietary information to make dietary choices, and awareness of the association between diet and disease. For the present study, a modified 12-item survey was created using items from the original scale. Participants were asked to decide whether or not they believed a health statement was true or false (e.g., “Butter is higher in calories than regular margarine”). Higher scores or more correct answers reflect better nutrition knowledge.

Choices. Participants were asked which beverages they would consume right now, given the choice, and how thirsty they were at the present time.

### 2.4. Analyses

Data was analyzed using SPSS. A Chi-squared test, correlational statistics, and a MANOVA were run.

## 3. Results

### 3.1. NFP Usage

Self-report of NFP usage was assessed in three different questions. Individuals could check yes or no to viewing the NFP in food packages in general, when they shop for food. Results showed that 61.1% (*n* = 159 of 260 participants who provided answers) affirmed using the NFP. In contrast, participants were less likely to report using the NFP in the current study (*n* = 100 of 258, 38.76%) (It is important to note that there are slight discrepancies in the total respondents for several questions, as not all participants answered all of the questions. Therefore the *n* value will vary slightly). This relatively low rate of NFP usage in the present study is surprising given that participants were encouraged to do so. Nevertheless, individuals in the No-Sugar condition reported using the NFP to evaluate the drinks at a higher rate (49.12%, *n* = 56 out of 114 individuals with no missing values) than those in the Sugar condition (30.5%, *n* = 44 of 144 individuals) (X² (1) = 9.24, *p* = 0.002). This is expected because the only way to judge content accurately would be from the label, but again we note the rates are not high. This result contrasts to no significance difference in reporting general NFP usage between the two groups (X² (1) = 0.184, *p* = 0.668).

The third assessment of NFP usage pertained to self-report frequency, or how often the participant states using the NFP when considering to purchase or consume a food item. A total of 151 participants (58.52%, *n* = 258), across both experimental conditions reported using the NFP 70% of the time or more. There were no differences in the pattern of responses to this question between the Sugar and No-Sugar individuals (X² (13) = 16, *p* = 0.249).

In terms of predicted relations of self-report of NFP usage with demographics, we found positive and significant correlations (all *p*s < 0.0001) with: nutrition knowledge (*r* = 0.325), education (*r* = 0.361), self-report of being healthy (*r* = 0.22), income (*r* = 0.25), self-report of eating healthy (*r* =0.5), eating regular meals (*r* = 0.46), being concerned with healthy eating (*r* = 0.56). Surprisingly, individuals asserting to use the NFP in general were less likely to admit using it in this particular study (*r* = −2, *p* = 0.001).

### 3.2. Accuracy of Sugar Estimates

The relationship (correlation) between the subjective and the objective amount of sugar in the beverages was examined in order to assess the degree to which individuals discriminated high versus low sugary drinks. This achievement measure derives from judgment analysis [39], a theory and methodology based on Brunswik’s lens model [40]. Using the number of teaspoons of sugar as the unit, Pearson’s correlation coefficient was computed for the judged and actual number of teaspoons of sugar across the 11 drinks for each person who had judged at least six drinks. As expected the judgment achievement of the Sugar group was higher (a median correlation equal to 0.6, which is strong and positive) than that of the No-sugar group with a median equal to 0.44. In other words, one half of the participants in the Sugar group had correlation equal to 0.6 or higher. Contrasting the mean correlation of the groups (mean *r* = 0.55 for the Sugar group, and mean *r* = 0.42 for the No-Sugar group), they are significantly different (using Fisher z transformation and unequal variance correction, t (244.36) = 3.48, *p* < 0.01). Thus, a simple display that makes the hidden sugar explicit allowed consumers to give estimates that more closely related to the actual amounts of sugar across the drinks. 

In terms of raw estimates of the number of sugar teaspoons, the proportion of sugar in each drink, and the amount of walking needed to burn the calories in the drink, the Sugar group tended to produce greater overestimation (greater error) in all cases. A MANOVA using the mean absolute error, computed as a difference between subjective and objective quantities (computed for each person) revealed a main effect of condition (F (3,251) = 2.72, Wilks’ Lambda = 0.97, *p* = 0.045) with larger means for the Sugar group with means equal to: 9.95 (SE = 1.89) for the teaspoon judgment; 40.04 min (SE = 3.47) for the walking judgment, and 28.58% for the proportion of sugar in the drink judgment (SE = 1.1). The corresponding means for the No-Sugar group were: 9.50 (SE = 2.15) for the teaspoon sugar judgment; 25.62 min for the walking estimate (SE = 3.95), and 26.68% for the proportion (SE = 1.26). 

We note that the differences were first computed; the sign of the average differences were positive for both groups but greater for the Sugar group (raw mean differences equal to 4.3 for the Sugar group and 2.06 for the No-sugar participants; the mean of the Sugar group is significantly larger (t (252) = 1.86, *p* = 0.03) (three outliers with means 2 standard deviations above the mean were removed for this test). The proportion of participants with positive means (displaying overestimation) was greater in the Sugar than the No-Sugar group (81 of 144, 56.25%, participants in the Sugar condition; 48 of 113, 42.47%, in the No-sugar condition), X^2^ (1) = 4.8, *p* = 0.028. In combination, using either the absolute or the raw differences, results point to greater overestimation by the Sugar than the No-Sugar group.

Comparing the judgments of healthiness of the beverages with the beverages’ NuVal showed similarity of the two groups. The median correlation for the Sugar group was 0.63 and that of the No-sugar group was equal to 0.61. Comparing the mean correlations resulted in no significant mean difference (mean correlations equal to 0.58 and 0.55, for the Sugar and No-sugar groups, respectively; *p* = 0.34). Thus, the sugar visualization did not affect judgments of nutrition quality of the drinks. Focusing on judgments errors with regards to judging healthiness, the groups did not differ either. However, absolute judgment errors in this variable tended to be smaller for individuals with higher nutrition knowledge (*r* = −124, *p* = 0.023) and education (*r* = −27, *p* = 0.00). Furthermore, individuals reporting higher nutrition knowledge also reported having better health (*r* = 0.134, *p* = 0.015) (all *p*s one-tail tests).

### 3.3. Participants with Diabetes

Focusing on participants who reported having diabetes (*n* = 43, 16.4%), this group had higher BMI (28.71) and lower income (median $20k and below annually) when compared to the rest of the participants (BMI = 26.24, median income $20–$29k annually). They were also older (median age 54; median age 46 for others). Results showed greater overestimation of sugar content by these individuals. The mean absolute error overestimating teaspoons of sugar was equal to 15.42 (with raw mean difference equal to 11.01). Without including the three outliers (one who was in the diabetics group), the mean of the absolute error describing overestimation for the diabetic group (mean = 9.9) is significantly larger than that of the rest of the participants (mean = 6.7), t (74.37) = 1.68, *p* = 0.049.

### 3.4. Person Level Factors that Relate to Judgment Accuracy

Regression analysis was employed to predict the accuracy measures from person-level characteristics. In particular, we hypothesized that nutrition knowledge and concern for healthy eating would result in greater accuracy. Because of the special health concern of diabetics, we also expected greater accuracy for this sub-group.

With regards to the correlation between the judged vs. objective total number of teaspoons in the drinks results showed that indeed the availability of sugar affected discrimination accuracy in the expected direction (*β* = 0.203, *p* < 0.01), but additionally individuals with higher levels of education and higher BMI had greater accuracy (*β* = 0.28, *p* < 0.01, for Education; *β* = 0.16, *p* < 0.01, for BMI; F (3, 242) = 12.62, *p* < 0.0001, adj R^2^ = 0.12). Surprisingly, higher nutrition knowledge, or higher concern for healthy eating did not predict this accuracy criterion. Of great interest is that individuals reporting having diabetes had no greater accuracy in judging relative sugar content than did individuals not having such a health issue. No other individual level variables were significant predictors of the relationship between subjective and objective amounts of sugar.

In terms of the average difference between judged and objective amounts of sugar (both in terms of proportion and of number of teaspoons), we found that nutrition knowledge was not predictive of these variables. In terms of the accuracy of judging amount of walking to be done to burn the calories, a model with condition, nutrition knowledge, diabetes and income as predictors resulted in, F (4, 240) = 2.72, *p* = 0.03, adj R^2^ = 0.027, but with significant beta weights for only the condition experimental manipulation (*β* = 0.17) with greater overestimation for those viewing the sugar display (i.e., the Sugar group).

Individuals with diabetes had greater overestimation of the amount of sugar present in the drinks as earlier stated. Additionally, the group of diabetics gave greater importance to sugar when judging the overall nutrition of foods (means = 51.63 and 42.83 for diabetics and controls, respectively). A MANOVA with both measures as dependent variables and group (diabetes vs. control) as independent variable revealed a significant group effect (F (2, 237) = 3.77, *p* = 0.024, Wilks’ Lambda = 0.97) (this analysis does not include the three outliers who produced very large estimates; results do not change when included).

### 3.5. Choice of Drink as a Function of the Visual Aid

The great majority of participants stated not wanting to consume any of the sugary drinks being judged at the moment (88.4% response rate towards not wanting to consume across participants and across the 10 drinks containing sugar). The condition manipulation, nevertheless, lowered the intentions of consuming any of the drinks; the mean number of drinks individuals felt like consuming was equal to 1.165 drinks for the No-Sugar group and equal to 0.89 for the Sugar group and this difference was statistically significant, t (255) = 2.077, *p* = 0.02 (one-tail). Another way to look at this is that in the No-Sugar group, across all participants and drinks, the average selection of sugary drinks was 14.9%, and this proportion was equal to only 8.8% in the Sugar group—a 40.93% decrease. These proportions are significantly different by z-test (*z* = 4.8 *p* < 0.0001).

We must note that on average people stated not being very thirsty with means equal to 39.33 and 36.89 for the Sugar and No-sugar groups, respectively, using the 0–100 scale with 100 denoting maximum thirst (these means are not statistically significantly different). Additionally, the open-ended question about drinks showed that the choices available in the study were not common drink options for participants. Orange juice was the most selected drink and this was stated by only 22 participants in the entire sample (8.4%). The next most popular drink was coke, but with only 5 selections. Besides these, water, coffee, and milk were more commonly listed as beverages consumers drink. Thus, the effect of the manipulation is likely to be stronger than observed if the individuals were thirstier and the sugary drinks were their habitual choices.

The results of this study also suggested that self-report of NFP usage both in general and for this study, as well as knowledge of nutrition and concern for healthy eating, did not play a significant role in predicting the total number of sugary drinks that participants reported they would hypothetically consume. However, it is important to note that these factors could be linked to habitual choices which would remain regardless of the intervention, and may be difficult to change. Other variables predictive of the choices, once the effect of the experimental manipulation was accounted for, were degree to which the person eats healthy (*β* = −18, *p* = 0.009), and education (*β* = −18, *p* = 0.006), F (5, 247) = 4.33, *p* < 0.0001, adj. R^2^ = 0.138.

Finally, we also found that in terms of estimating sugar in drinks, individuals were generally off, overestimating sugar by three spoons or more. This was true even for individuals with higher levels of education, nutrition knowledge, and concern for healthy eating. The simplest explanation is that, in general, people have no concept of the correlation between grams (the measure on the NFP) and teaspoons. Grams, for US participants, is also a more abstract concept. This would suggest that the NFP is of little benefit whether or not it is used, in helping to determine overall quantities of sugar. Strong positive correlations among self-report of NFP usage in daily life with concerns for eating healthy, education, income, nutrition knowledge suggests a “wealthier get wealthier” scenario, in that those who are aware of the value of, and are concerned with, health knowledge, are better prepared to make nutritional judgments than those who are not. This will be further elaborated on in the discussion.

## 4. Discussion

The evidence from medical and health care research is mounting to support the link between sugar consumption and cardiovascular disease and mortality [41]. The politics behind the high availability of sugary drinks and food products containing added sugars is complex [42]. In the center of these realities lies the psychological machinery that reacts positively to sugar and does not perceive the world in a purely objective way. It is the judgment and decision-making processes that ultimately determine the degree to which consumers are able to judge information effectively and use it to make smart food selections. 

Our work focuses on understanding the psychological judgment processes with the hope that interventions, other than those based on pricing and/or availability of products, can be developed to support effective decision making. How well can individuals judge how much of a nutrient is present in a food product? What factors contribute to accurate perceptions and cognitions? Answers to these questions, we believe, are essential in determining support systems that result in calibrated perceptions and more optimal food choices.

The study was conducted mostly in rural Appalachia, but it also had individuals from a community center in Cleveland which allows our results to generalize to a range of income and education. We tested a simple intervention designed to make sugar explicit when considering amounts of sugar in a set of popular drinks. Two important findings from this study are: 1) estimation of nutrient content was difficult even when sugar amounts were made obvious via the test-tube sugar displays for each drink, and 2) both judgments and choices were influenced by the intervention. With a few exceptions, other person level characteristics, such as nutrition knowledge and concern for healthy eating, did not influence judgment accuracy.

In terms of estimating sugar content, such as number of teaspoons of sugar, individuals were better able to discriminate among drinks when sugar was made explicit. Additionally, higher levels of education and higher BMI related to higher accuracy, but contrary to our expectation, no relationship was found with nutrition knowledge and concern for healthy eating. Exact estimates, on the other hand, were no more accurate, but tended to move in the direction of overestimation. The overestimation also occurred with regards to amount of time walking needed to burn the calories.

From the perspective of the helpfulness of the NFP information we note that sugar amounts, as described in the label, did not translate into common units such as teaspoons, and individuals were generally off, overestimating sugar by three spoons or more. This was true even for individuals with higher levels of education, nutrition knowledge, and concern for healthy eating. Of great consequence is the fact that we found strong positive correlations among self-report of NFP usage in daily life with concerns for eating healthy, education, income, nutrition knowledge. However, NFP frequency related negatively to using the NFP in the current study and it did not predict judgment accuracy of any type, nor did it predict choice. Thus, our results cast doubts on the meaning and validity of high levels of NFP usage derived from self-report.

On the positive side, whatever information was used from the label, or from past experience, the judgments about the overall nutritional quality of the drinks produced relatively high discriminations as measured by the correlation between NuVal (the objective nutrition scores) and the subjective impressions. Accuracy with respect to NuVal also depended on nutrition knowledge and education.

In terms of drink selection, we found a low rate of preference for the options the study provided, yet the visual aid manipulation influenced choice. Using the total number of sugary-drinks a participant may drink as a measure of consumption intention, we found that the visual displayed produced lower rates of consumption. Beyond this manipulation effect, self-report of eating healthy and education were the only other predictors of choices. Interestingly, the status of being diabetic, having concern for healthy eating, or identifying sugar as an important nutrient did not predict choice.

Focusing on the group of 43 diabetics across locations, we noted that they reported giving great importance to sugar when judging nutrition as would be expected. In addition, their estimates of sugar content were greater than the rest of the participants by an average of approximately three teaspoons. But the group did not differ in terms of drink selections, as previously mentioned, which highlights the possible disconnect between beliefs and actions.

Finally, another interesting finding was that individuals who reported that they regularly used the NFP were less likely to admit to using it in the present study. As this did not vary as a function of condition (e.g., they were not more or less likely to use the NFP if the sugar tubes were present), other factors could be at work. More research is needed to determine if this was simply a function of being part of a research study, or their normal habitual behavior.

It is also possible that these individuals in general tended to adhere to the social desirability bias, and thus wanted to report that using the NFP was a regular habit, as it was the center of the study and known to be beneficial. They may also want to use it, but in general tend to forget, or get distracted when they do.

## 5. Conclusions

In the sugar debate, the psychology of sugar needs greater attention with emphasis on the perceptual and cognitive processes that determine judgments and choices. The human perception system is not a purely bottom up information processor reflecting objective quantities, and judgments are influenced as much from expectations and suggestions as they are from the sensory processes from which those judgments come from [43]. Greater attention to these psychological underpinnings is in demand in order to progress towards creating environments that support effective choices. Such environments may need to go beyond placing limits on food availability via pricing, or the lowering of supply, which present implementation challenges. Our findings demonstrate that nutrient visualization can support judgments and decisions and thus may be a viable tool for curtailing consumption of undesirable nutrients. Perhaps, labels that more obviously convey information, such as providing the exact number of teaspoons of sugar in the product, are in demand.

## Figures and Tables

**Table 1:** Sample Size in Different Locations by Experimental Condition.

Location	Sugar	No-sugar	Total
Lottridge Ridge Food Center	25	23	48
Save-A-Lot	38	41	79
Athens Farmers Market	42	21	63
Solon Community Center	41	30	71
Total	146	115	261

**Table 2:** Study Drinks with Key Information.

Drink Name	NuVal Score	Serving Size (Ounces)	Serving Per Container	Calories Per Serving	Total Calories Per Bottle	Total Sugar (Grams) Per Bottle	Number of Teaspoons Sugar (1 tsp = 4 g)	Number of Sugar Bottles	Minutes to Burn Calories
Organic Horizon Low-Fat Chocolate Milk	32	8	1	150	150	22	5.5	1.375	30
Pepsi	1	12	1	150	150	41	10.25	2.5625	30
Monster Energy Drink	3	8	2	110	220	54	13.5	3.375	44
Starbucks Frappuccino Mocha (Low-Fat)	23	9.5	1	180	180	31	7.75	2	36
Diet Snapple Lemonade Iced Tea Half n’ Half	40	16	1	10	10	0	0	0	2
Coca Cola	1	12	1	140	140	39	9.75	2.5	28
Odwalla Mango Tango Fruit Smoothie Blend	31	12	1	220	220	44	11	2.75	44
Sprite Lemon Lime Soda	1	12	1	140	140	38	9.5	2.375	28
Simply Orange (Florida’ s Natural 100% Orange Juice)	30	13.5	1	190	190	41	10.25	2.5625	38
Red Bull Cola	1	8.4	1	110	110	27	6.75	1.6875	22
Gatorade Lemon-Lime G2 Thirst Quencher	1	12	2.5	30	75	17.5	4.375	1.09375	15

tsp = teaspoons.

## Supplementary Materials

The following are available online at https://www.mdpi.com/2072-6643/12/2/394/s1, Figure S1: Questions About Drinks; Figure S2: demographic questionnaire; Figure S3: Nutrition Questions; Figure S4: Overall Questions
