# Supplementary material for: How Much Sugar is in My Drink? The Power of Visual Cues
Source: Nutrients. 2020 Feb 2;12(2):394. doi: 10.3390/nu12020394 (PMC7071271; doi:10.3390/nu12020394)
Supplement: Supplementary file 1 [file nutrients-12-00394-s001.pdf]

Supplementary Materials

Please answer the following questions based on what you observed today at the nutrition and sugar display.

Item #1: Organic Horizon Low-fat Chocolate Milk

1.) If you consume chocolate milk, **how many times a week** do you drink it (put 0 if you never consume it or don't like it) ______?

2.) What **proportion** of this beverage is sugar (e.g., if a drink contains ½ a cup of sugar, and ½ cup of milk, the beverage would be ½ or 50% sugar)?

Organic Horizon Low-fat Chocolate Milk is ______% Sugar

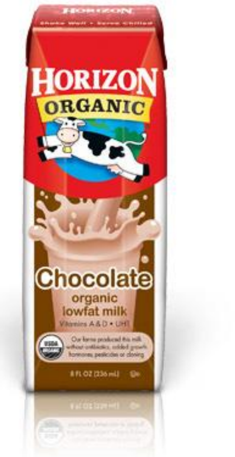

	Not Healthy				Somewhat Healthy					Extremely Healthy	
3.) How HEALTHY is this beverage?	0	10	20	30	40	50	60	70	80	90	100

	Does Not Meet Them				Meets Some Requirements					Meets them Extremely Well	
4.) How well does the beverage meet nutritional requirements/how NUTRITIOUS is the drink?	0	10	20	30	40	50	60	70	80	90	100

5.) How many teaspoons of sugar are in this drink? ______teaspoons

6.) How many minutes of brisk walking (3.5 mph) would it take to burn off the calories from consuming this drink (assume you are drinking the **ENTIRE** bottle, which may contain more than one serving size)?

______ minutes

7.) How confident are you in your answer?

50%	60%	70%	80%	90%	100%
just guessing					absolutely sure

Figure S1. Questions About Drinks.

Please answer each question as accurately and truthfully as possible. ALL responses are ANONYMOUS and confidential - there will be no way to link your responses to these questions back to you.

Thank you again so much for your time and help!!

1.) What is your height in feet and inches (e.g., 5'6")?
   ______feet _____inches
2.) What is your weight in pounds? ______ lbs
3.) What is your age? _______
4.) What is your gender (circle one)? Male Female
5.) What is the highest level of education you have completed?
   a. Less than High School
   b. High School/GED
   c. Some College
   d. 2-year College Degree
   e. 4-year College Degree
   f. Master's Degree
   g. Doctoral Degree
   h. Professional Degree (JD, MD)
6.) What is your employment status?
   a. Student
   b. Employed
   c. Not employed
   d. Disability
   e. Homemaker
   f. Retired
   g. Other _______
7.) Are you currently dieting? YES NO
8.) Do you follow a particular diet (circle one)?
   a. N/A
   b. Weight Watchers, Mediterranean diet, Paleolithic diet, Vegetarian, Vegan, Atkins diet, South Beach, Zone diet, Dr. Oz diet.
   c. Other:_________
9.) What it your annual income range?
   a. Below $20,000
   b. $20,000 - $29,999
   c. $30,000 - $39,999
   d. $40,000 - $49,999
   e. $50,000 - $59,999
   f. $60,000 - $69,999
   g. $70,000 - $79,999
   h. $80,000 - $89,999
   i. $90,000 or more
10.) What is your race?
   a. White/Caucasian
   b. African American
   c. Hispanic
   d. Asian
   e. Native American
   f. Pacific Islander
   g. Other_________

Please Circle Your Answer Below:

	Never					Half the time				All the Time	
11.) Do you snack?	0%	10	20	30	40	50%	60	70	80	90	100%
12.) Do you eat healthy foods?	0%	10	20	30	40	50%	60	70	80	90	100%
13.) Do you have regular meals (breakfast, lunch, dinner)?	0%	10	20	30	40	50%	60	70	80	90	100%
14.) Are you concerned with healthy eating?	0%	10	20	30	40	50%	60	70	80	90	100%
15.) How often do you use the Nutrition Facts Panel when you consider whether to purchase or consume a food item?	0%	10	20	30	40	50%	60	70	80	90	100%

15.) How often do you eat meals outside of your home (delivery, carryout, or dining out)?
   a. Never
   b. Less than once a month
   c. 1-3 times a month
   d. Once a week
   e. 2-3 times a week
   f. 4-5 times a week
   g. More than 5 times a week

16.) Do you have health issues or other dietary/religious restrictions that affect your food choices (e.g., diabetes, allergies)?

YES NO

17.) If YES, please circle all that apply:
   a. Diabetes/pre-diabetic
   b. Allergies
   c. High blood pressure
   d. High blood cholesterol
   e. High blood triglycerides
   f. Genetic disorder
   g. Religious restrictions
   h. Vegan
   i. Vegetarian
   j. Gluten intolerant
   k. Other: Please describe: ______________________

Please Circle Your Answer Below:

	Sick, very unhealthy									Perfect Health	
18.) Overall, how healthy do you believe you are at the present time?	0	10	20	30	40	50	60	70	80	90	100

19.) What information on a food package do you tend to read/look for (choose all that apply)?

a. Health claims (e.g., 100% whole grains, low sodium, American Heart Association (AHA) approved)
b. Ingredients
c. Nutrition Facts Panel
d. GMO (genetically modified organism)
e. Organic
f. Local
g. Picture/image of item
h. Games/entertainment
i. Expiration data
j. Instructions for consumption/use
k. other _____

20.) If you use the Nutrition Facts Panel when evaluating a product, which nutrient information do you consider to be **the most important** in making your decision?

Please **RANK** the following with 1 = most important to 14 = least important. If you don't use the Nutrient Facts Panel, please select the information you think should be the most important in making a decision. **PLEASE USE EACH NUMBER ONLY ONCE!**

____calories
____calories from fat
____total fat
____saturated fat
____trans fat
____poly/monounsaturated fats
____carbohydrates
____cholesterol
____sodium
____fiber
____sugar
____protein
____vitamins
____additives (e.g., BHT, Carmel color, food coloring, hydrogenated oils)

21.) Did you use the Nutrition Facts Panel when evaluating food items in **THIS** study?

YES NO

22.) If you used the Nutrition Facts Panel in **THIS** study, did you focus on some nutrients more than others?

If so, please **RANK** the importance of the nutrients you **focused on for this study** from 1 = most important to 14 = least important. If you did not use the Nutrient Facts Panel, please select the information you think should be the most important in making a decision for the items in THIS study. **PLEASE USE EACH NUMBER ONLY ONCE!**

____calories
____calories from fat
____total fat
____saturated fat
____trans fat
____poly/monounsaturated fats
____carbohydrates
____cholesterol

____sodium
____fiber
____sugar
____protein
____vitamins
____additives (e.g., BHT, Carmel color, food coloring, hydrogenated oils)

23.) What do you believe the purpose of this study was?

____

____

____

24.) What do you believe would have the greatest impact on your decision to purchase a food or drink item (please select all that apply)?

a. Hunger/thirst at the time.
b. Price reduction (e.g., Low sugar items are 15% off).
c. Price Bonus (e.g., get an extra amount of money for each healthy item you purchase).
d. Visual educational material (e.g., display showing how much sugar or fat is contained in an item).
e. Nutritional education material (e.g., display with experts talking about the negative health effects of sugar).
f. Information on how many calories you would have to burn if you consumed the item (e.g., you would have to walk for 40 min to burn off the calories consumed in one serving of Kashi Strawberry Fields cereal).

Other____

____

____

Figure S2. demographic questionnaire.

1. Experts advise that people eat 3 servings of fruit and vegetables a day (One serving could be, for example, an apple or a handful of chopped carrots).

[True / False]

2. The most important fat for people to cut down on is monounsaturated fat.

[True / False]

3. Some foods contain a lot of fat but no cholesterol.

[True / False]

4. A glass of unsweetened fruit juice counts as a helping of fruit.

[True / False]

5. Saturated fats are mainly found in dairy products.

[True / False]

6. Brown sugar is a healthy alternative to white sugar.

[True / False]

7. There is more protein in a glass of whole milk than in a glass of skimmed milk.

[True / False]

8. Polyunsaturated margarine contains less fat than butter.

[True / False]

9. White bread contains more vitamins and minerals than brown or whole grain bread.

[True / False]

10. Butter is higher in calories than regular margarine.

[True / False]

11. Coconut oil contains mostly monounsaturated fat.

[True / False]

12. Polyunsaturated fats are mainly found in vegetable oils.

[True / False]

Figure S3. Nutrition Questions

1.) If you could have any of these drinks to consume RIGHT NOW, which would you choose (please circle all that apply)?

a. NONE, I do not like, consume or purchase any of these beverages.
b. Organic Horizon Low-fat Chocolate Milk
c. Pepsi
d. Monster Energy Drink
e. Starbucks Frappuccino Mocha (Low-fat)
f. Diet Snapple Lemonade Iced Tea Half n' Half
g. Coca Cola
h. Odwalla Mango Tango Fruit Smoothie Blend
i. Sprite
j. Simply Orange Juice
k. Red Bull
l. Gatorade Lemon-Lime G2 Thirst Quencher

2.) How many POUNDS per year would you gain if you consumed one extra can of a soft drink or other sugary beverage (e.g., 10 teaspoons of sugar, 150 calories) a day?

______pounds

3.) How many teaspoons of sugar should you consume per day based on the 2000 calorie guidelines?

_______ teaspoons

	Not thirsty at all									Parched, dying of thirst	
4.) How thirsty are you RIGHT NOW?	0	10	20	30	40	50	60	70	80	90	100

5.) Which drinks shown here are the healthiest? Please rank the drinks from 1 = Healthiest to 11 = Least Healthy. **PLEASE USE EACH NUMBER ONLY ONCE!**

____Organic Horizon Low-fat Chocolate Milk)
____Pepsi
____Monster Energy Drink
____Starbucks Frappuccino Mocha (Low-fat)
____Diet Snapple Lemonade Iced Tea Half n' Half
____Coca Cola
____Odwalla Mango Tango Fruit Smoothie Blend
____Sprite
____Simply Orange Juice
____Red Bull
____Gatorade Lemon-Lime G2 Thirst Quencher

	Not at all important								Most important nutrient to consider		
6.) How important is sugar to an items overall nutrition?	0	10	20	30	40	50	60	70	80	90	100

7.) What beverages do you most often consume, and how often do you consume them during a week (e.g., Orange Juice, 6 glasses a week)?

____

Please View the Following Information and then Answer **TWO** Questions

PEPSICO

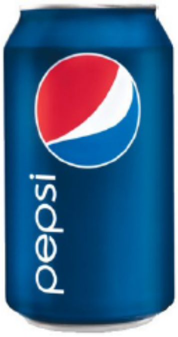

Pepsi

Type: Bottles, Cans and Cartons **Size:** 12 fl oz

Nutrition Info:

Serving size 1 container	Per Container	
	12 fl oz	%DV*
Calories	150	-
Total Fat (g)	0	0
Sodium (mg)	30	1
Total Carbs (g)	41	14
Sugars (g)	41	-
Protein (g)	0	-

Not a significant source of other nutrients.
*Percent Daily Values (DV) are based on a 2,000 calorie diet.

Calorie and nutrient values are rounded as required by the Food & Drug Administration. This can produce irregularities among sizes. Product may not be available in all areas.

Ingredients:
CARBONATED WATER, HIGH FRUCTOSE CORN SYRUP, CARAMEL COLOR, SUGAR, PHOSPHORIC ACID, CAFFEINE, CITRIC ACID, NATURAL FLAVOR

www.pepsicobeveragefacts.com
Last Updated May 24, 2013

PEPSICO

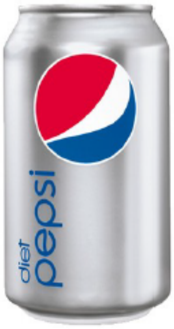

Diet Pepsi

Type: Bottles, Cans and Cartons **Size:** 12 fl oz

Nutrition Info:

Serving size 1 container	Per Container	
	12 fl oz	%DV*
Calories	0	-
Total Fat (g)	0	0
Sodium (mg)	35	2
Total Carbs (g)	0	0
Sugars (g)	0	-
Protein (g)	0	0

Not a significant source of other nutrients.
*Percent Daily Values (DV) are based on a 2,000 calorie diet.

Calorie and nutrient values are rounded as required by the Food & Drug Administration. This can produce irregularities among sizes. Product may not be available in all areas.

Ingredients:
CARBONATED WATER, CARAMEL COLOR, ASPARTAME, PHOSPHORIC ACID, POTASSIUM BENZOATE (PRESERVES FRESHNESS), CAFFEINE, CITRIC ACID, NATURAL FLAVOR, ACESULFAME POTASSIUM

8.) Which of these items is the HEALTHIER choice?
Pepsi Diet Pepsi

9.) Which is lower in SUGAR?
Pepsi Diet Pepsi

Figure S4. Overall Questions.
